# Transcranial stimulation combined with four rehabilitation therapies for gait and motor function in Parkinson’s disease: a network meta-analysis of 23 RCTs

**DOI:** 10.3389/fnagi.2025.1670825

**Published:** 2025-12-15

**Authors:** Dongyue Li, Xinyu Lin, Haojie Li, Jian Zhou

**Affiliations:** 1 School of Physical Education, Guangzhou University, Guangzhou, China; 2 School of Exercise and Health, Shanghai University of Sport, Shanghai, China

**Keywords:** Parkinson, rehabilitation, transcranial stimulation, gait, motor, combined exercise therapy

## Abstract

**Introduction:**

Parkinson’s disease (PD) has become the fastest-growing neurological disease worldwide. This network meta-analysis evaluated the efficacy of transcranial stimulation combined with four rehabilitation approaches for improving gait and motor function in Parkinson’s disease.

**Methods:**

We systematically searched seven databases: PubMed, Embase, Cochrane Library, Web of Science, CNKI, and Wanfang. Data from 23 randomized controlled trials (*n* = 669 patients) were analyzed using a frequentist network meta-analysis approach. Primary outcomes included gait parameters (velocity, cadence, stride length) and motor function (Timed Up and Go test, Unified Parkinson’s Disease Rating Scale Part III). Statistical analyses incorporated the Surface Under the Cumulative Ranking curve rankings and sensitivity analyses.

**Results:**

(1) For gait outcomes, Dual-Task Training showed optimal efficacy for improving stride length (SUCRA = 100%) and velocity (86.5%), while Exercise Rehabilitation best improved cadence (100%). (2) For motor function, Conventional Rehabilitation demonstrated superior improvement in the Timed Up and Go test (100%), and Dual-Task Training showed advantages in Unified Parkinson’s Disease Rating Scale Part III scores (85.1%). All combined interventions significantly outperformed the control groups (*p* < 0.05), and sensitivity analyses confirmed the robustness of these findings.

**Conclusion:**

The results support the use of personalized rehabilitation strategies: Dual-Task Training for patients with stride deficits and prominent motor symptoms, Exercise Rehabilitation for cadence improvement, and Conventional Rehabilitation for enhancing general mobility. These findings provide evidence-based guidance for optimizing neurorehabilitation protocols in the management of Parkinson’s disease.

## 1 Introduction

Parkinson’s disease (PD) has become the fastest-growing neurological disorder worldwide (Pringsheim et al., 2014). Driven by population aging, the global number of PD patients is projected to increase from 6 million in 2016 to 12 million by 2040 (GBD 2016 Parkinson's Disease Collaborators, 2018). As a predominantly motor disorder, PD is characterized by progressive motor dysfunction, including gait impairment, bradykinesia, and postural instability (Gao et al., 2020; Lin et al., 2021; Grimbergen et al., 2009). Notably, gait disturbances (e.g., freezing of gait, step shortening, and asymmetry) are directly associated with an annual fall rate of up to 68% (Kim et al., 2022). Given the limited efficacy of pharmacological treatments for these persistent functional deficits, rehabilitation remains a cornerstone for functional recovery in PD management.

Current standard rehabilitation strategies for PD—such as physical therapy, treadmill training, and external cueing techniques—primarily rely on task-specific training and compensatory mechanisms to improve motor function, yet their clinical utility faces significant limitations (Gulcan et al., 2023; Robinson et al., 2019; Nascimento et al., 2024). Studies indicate that PD’s neurodegenerative nature substantially restricts neuroplasticity, thereby impeding training-induced neural reorganization (Johansson et al., 2020).

Transcranial Stimulation techniques, principally transcranial direct current stimulation (tDCS) and repetitive transcranial magnetic stimulation (rTMS), enhance conventional rehabilitation through distinct yet complementary mechanisms (Broeder et al., 2023; Agarwal et al., 2019). These techniques differ fundamentally in their mode of action: tDCS applies a low-intensity electrical current to modulate the resting membrane potential of neurons, thereby priming the cortex for enhanced neuroplasticity (Zaghi et al., 2010). In contrast, rTMS uses electromagnetic induction to generate focused currents that can depolarize neurons, inducing synaptic plasticity through mechanisms akin to long-term potentiation or depression (Che et al., 2021). Despite these mechanistic differences, both modalities share the capacity to modulate cortical excitability. When applied to regions such as the primary motor cortex, they can synergistically augment training-induced neural oscillatory coupling, leading to improved functional outcomes (Reis et al., 2009).

Despite mechanistic support for combined therapies, the relative efficacy of different protocols lacks systematic evaluation (Nguyen et al., 2024; Lee et al., 2025). Existing studies predominantly focus on single interventions, failing to directly compare multiple approaches, while traditional meta-analyses cannot rank treatment efficacy (Li et al., 2022; Elsner et al., 2016; Zhu et al., 2015; Shen et al., 2016). This network meta-analysis (NMA) will systematically evaluate and rank four rehabilitation therapies augmented with transcranial stimulation for PD, providing evidence-based guidance for optimal treatment selection and informing clinical decisions to improve functional outcomes in PD patients.

## 2 Methods

### 2.1 Protocol and registration

This study was conducted in strict accordance with the PRISMA-NMA guidelines (Preferred Reporting Items for Systematic Reviews and Network Meta-Analyses). The study protocol was registered with PROSPERO, the International Prospective Register of Systematic Reviews (Registration ID: CRD420251106424). The aim of this study was to compare the efficacy of different rehabilitation exercises combined with Transcranial Stimulation in improving walking ability and motor function in patients with Parkinson’s disease, using a network meta-analysis. Key analyses included pooled effect sizes for direct and indirect comparisons, inconsistency testing, and intervention ranking.

### 2.2 Eligibility criteria

Inclusion criteria:

- Participants: patients with idiopathic Parkinson’s disease meeting UKPDSBB diagnostic criteria, age ≥18 years, Hoehn-Yahr stage I-III.
- Interventions: rTMS or tDCS combined with Conventional Rehabilitation (CR), Exercise Rehabilitation (ER), Feedback Training (FT) or Dual-Task Training (DTT) in the experimental group, and Rehabilitation Training Alone/Shock Stimulation Combined with Rehabilitation Training/Transcranial Stimulation Alone in the control group.
- Study Design: Randomized controlled trial (RCT) with parallel or crossover design.
- Outcomes: Walking function including step speed [m/s], step frequency [steps/min] and stride length [m]; motor function including Unified Parkinson’s Disease Rating Scale, Part III (UPDRS-III) and Timed Up and Go (TUG) test.

Exclusion criteria:

- Comorbidities with other neurologic diseases such as Alzheimer’s disease, stroke, etc.
- Diagnosis of secondary Parkinsonism, Parkinson-plus syndromes, or hereditary degenerative Parkinsonian disorders.
- Those receiving invasive treatments such as deep brain stimulation.
- Inability to extract key data such as mean and standard deviation.
- Incomplete studies such as conference abstracts, case reports, etc.

### 2.3 Search strategy

PubMed, Embase, Cochrane Library, Web of Science, CNKI and Wanfang databases were systematically searched to ensure comprehensive coverage of published relevant studies. The timeframe of the search was set from the establishment of each database to May 31, 2025.

The search strategy was designed based on the PICOS framework, and a combination of subject terms (MeSH) and free words was used to construct the search formula. For example, search terms used in PubMed included rTMS, tDCS, PD, Motor function, gait function, and RCT are key terms.

- Participants: “Parkinson Disease” [Mesh], “Parkinsonian Disorders” [Mesh].
- Interventions: “Transcranial Magnetic Stimulation” [Mesh], “Transcranial Direct Current Stimulation” [Mesh], “Non-Invasive Brain Stimulation”[Mesh], “Physical Therapy Modalities”[Mesh], “Exercise Therapy”[Mesh].
- Outcomes: “Gait”[Mesh], “Walking Speed”[Mesh], “Motor Activity”[Mesh], “Unified Parkinson Disease Rating Scale”[Mesh].
- Study Design: “Randomized Controlled Trial”[Publication Type].

To ensure a complete literature search, we manually screened the references of the included studies. Ultimately, only studies published in English and Chinese were included to reduce language bias.

### 2.4 Study selection and data extraction

Two researchers independently performed literature screening. Firstly, using EndNote 20 software to remove duplicates, followed by initial screening by title and abstract, then full-text review with any disagreements resolved through group discussion or third researcher arbitration. Data extraction included (1) study characteristics: first author, year of publication, country, study design, and duration; (2) patient characteristics: sample size, age, sex ratio, and duration of disease; (3) intervention details: treatment cycles and combined training programs; and (4) outcome data: Gait velocity, Cadence, and Stride Length were all derived from biomechanical automatic collection equipment, and if Gait velocity was reported from separate walking tests, its units were unified before analysis; the mean and standard deviation of TUG and UPDRS-III at baseline and endpoint, or the mean and standard deviation of their changes before and after intervention, were directly extracted; all extractions followed the principle of prioritizing baseline and endpoint data, with change value data used when the former was unavailable, to ensure analysis reliability.

### 2.5 Risk of bias assessment

In this study, we rigorously assessed the risk of bias in included studies using the Cochrane RoB 2.0 tool, which evaluates five core domains: (1) selection bias (random sequence generation and allocation concealment); (2) performance bias; (3) detection bias; (4) attrition bias; and (5) reporting bias. Each domain is judged as follows:

- Low risk: means the study has a very low likelihood of bias in that area;
- Some concerns: means that the study may have some bias in that area but it is uncertain;
- High risk: where the study is at definite risk of bias in this area.

Two investigators independently conducted the assessments, and the Kappa statistic was used to assess inter-rater agreement (Kappa > 0.8 was considered good agreement), and disagreements were resolved through group discussion or arbitration by a third investigator. Studies with some concerns in 2 domains were considered at moderate risk, and those with some concerns in >2 domains were considered at high risk. For crossover design studies in particular, we additionally evaluated the adequacy of washout periods to ensure inter-study comparability and reliability of results.

### 2.6 Statistical analysis

In this study, Standardized Mean Difference (SMD) and its 95% Confidence Interval (95% CI) were used as the effect size measures for continuous outcomes. For the statistical analysis framework, a random-effects model was employed based on the frequentist approach to fully consider the potential clinical and methodological heterogeneity among studies. To address the unique inconsistency issues in network meta-analysis, the study used the node-splitting approach for local inconsistency testing, and the design-by-treatment interaction model combined with the Wald test to assess the global inconsistency. The Heterogeneity was assessed using the I^2^ statistic (with a threshold of >50%) for quantitative analysis. To comprehensively evaluate the relative efficacy ranking of interventions, the study calculated the Surface Under the Cumulative Ranking Probability Curve (SUCRA, with a value range of 0–100%), which was positively correlated with the ranking of interventions. In addition, the potential influence of disease severity (based on the Hoehn-Yahr staging system) and treatment duration on effect sizes was focused through a predefined subgroup analysis strategy. Funnel plots with Egger’s regression test was used to assess potential publication bias. All statistical analyses were performed using professional statistical software, including Stata 17.0 (network package) and RevMan 5.4, to ensure the methodological standardization of the analyses and the reliability of the results. This paragraph clearly states that all gait velocity data were converted to a uniform unit (m/s) prior to analysis, and that the SMD was calculated based on these standardized values.

## 3 Result

### 3.1 Study selection

Through systematic searches, we initially identified 1,856 potentially relevant articles. After automated deduplication using EndNote 20 software and manual verification, 865 duplicate records were removed, leaving 991 articles for screening. Two researchers independently screened titles and abstracts based on predefined inclusion/exclusion criteria (following PICOS principles), excluding 903 clearly ineligible articles (including 357 with incompatible interventions, 389 with ineligible participants, and 157 with inappropriate study designs).

**Figure 1:**
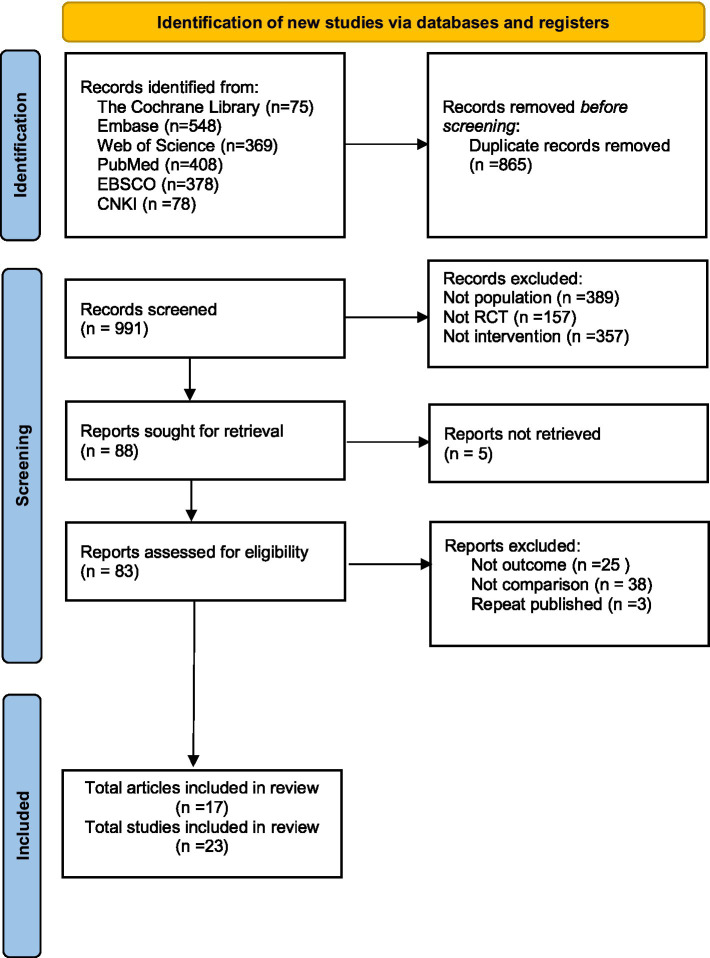
Flowchart of study selection.

The remaining 88 articles underwent full-text retrieval and detailed evaluation, ultimately yielding 17 articles comprising 23 eligible randomized controlled trials. Any discrepancies during the screening process were resolved through arbitration by a third researcher. The complete literature selection process and reasons for exclusion were documented in a PRISMA-compliant flow diagram (Figure 1), ensuring full traceability and transparency of the study selection procedure.

### 3.2 Study characteristics

This study systematically analyzed 23 clinical studies (see Appendix 1 for details), involving a total of 669 participants. Demographic characteristics showed a mean age of 65.97 years, with female participants accounting for 46.2%. The included studies investigated four main intervention approaches: conventional rehabilitation (CR, 9 studies), exercise-based rehabilitation (ER, 6 studies), functional training (FT, 5 studies), and task-oriented training (DTT, 3 studies), comprehensively covering current mainstream rehabilitation protocols.

### 3.3 Network meta-analysis

**Figure 2:**
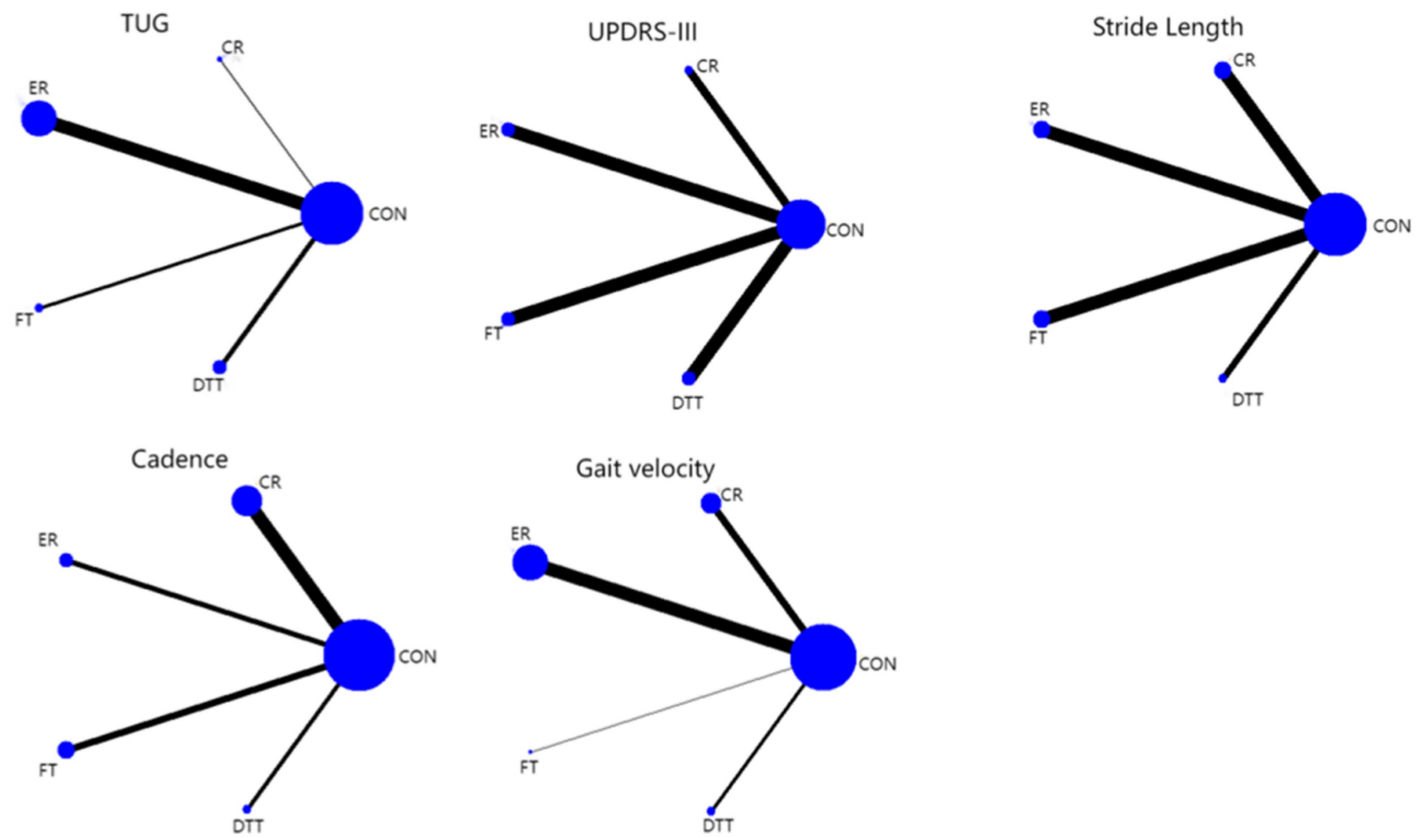
Network diagram of outcome measures.

The network plot (Figure 2) comprised five intervention arms: CR, ER, FT, DTT, and control (CON). Node sizes represented the sample sizes of respective interventions, while edge thickness reflected the number of studies available for direct comparisons. All interventions were interconnected through either direct or indirect evidence.

#### 3.3.1 Direct comparison results

The results of pairwise meta-analysis are presented in Appendix 2.

##### 3.3.1.1 For gait velocity

CR (1.47, 95% CI 0.49–2.45), ER (1.21, 0.38–2.04), and DTT (1.28, 0.63–1.94) all showed significant improvements compared to CON, while FT (0.87, −0.01–1.75) did not reach statistical significance. This indicates that all three interventions except FT were superior to the control group in improving gait speed, with CR showing the largest effect size.

##### 3.3.1.2 For cadence

CR (1.73, 1.02–2.44), ER (4.74, 1.71–7.77), and FT (1.26, 0.00–2.51) all demonstrated significant improvements compared to CON, with ER showing the most prominent effect. It should be noted that although the lower limit of FT’s confidence interval was 0, it was still marked as significant. DTT (0.38, −0.32–1.08) showed no significant effect, suggesting it may be less effective than other interventions in improving step frequency.

##### 3.3.1.3 For stride length

All interventions showed significant improvements: CR (0.85, −0.36–2.06), ER (5.44, 1.57–9.30), FT (16.25, 9.88–22.63), and DTT (11.02, 3.08–18.96). Notably, FT and DTT showed exceptionally high effect sizes with wide confidence intervals, suggesting substantial individual differences in their effects on stride length improvement.

##### 3.3.1.4 For TUG test

CR (11.22, 9.57–12.87) showed extremely large effect sizes with very precise estimates. Although ER (1.67, −0.11–3.46), FT (0.11, −2.21–2.43), and DTT (1.26, −2.95–5.47) were marked as significant, their confidence intervals either included null values (ER), approached null values (FT), or were extremely wide (DTT), requiring cautious interpretation of these results’ clinical significance.

##### 3.3.1.5 For UPDRS-III scores

CR (0.80, 0.18–1.42) and ER (0.92, 0.43–1.42) showed moderate but stable improvements. DTT (1.80, 0.16–3.45) had a large effect size but imprecise estimates. Although FT (29.21, −2.54–60.96) was marked as significant, its extremely wide confidence interval that included negative values suggests this anomalous result may require further validation, possibly due to data abnormalities or extreme values.

#### 3.3.2 Network comparison results

##### 3.3.2.1 Gait function

*For Gait velocity*: DTT showed significant advantage versus CON (SMD −1.28, 95%CI −1.92 to −0.64), ER was also statistically significant versus CON (SMD −1.00, 95%CI −1.27 to −0.73), and CR showed significant difference versus CON (SMD −0.79, 95%CI −1.04 to −0.54). In direct comparisons between interventions, no significant differences were found between DTT and ER (SMD −0.28, 95%CI −0.97 to 0.41), DTT and FT (SMD −0.41, 95%CI −1.50 to 0.68), or DTT and CR (SMD −0.49, 95%CI −1.17 to 0.20). Similarly, no significant differences were found between ER and FT (SMD −0.13, 95%CI −1.05 to 0.79), ER and CR (SMD −0.21, 95%CI −0.57 to 0.16), or FT and CR (SMD −0.08, 95%CI −0.99 to 0.84). Notably, FT versus CON approached statistical significance (SMD −0.87, 95%CI −1.75 to 0.01).

*For cadence*: ER showed optimal effects versus CON (SMD −3.33, 95%CI −4.01 to −2.65), followed by CR (SMD −1.77, 95%CI −2.08 to −1.46) and FT (SMD −1.28, 95%CI −1.66 to −0.90). In direct comparisons between interventions, ER significantly outperformed CR (SMD −1.56, 95%CI −2.31 to −0.81), FT (SMD −2.04, 95%CI −2.82 to −1.26), and DTT (SMD −2.94, 95%CI −3.92 to −1.97). CR versus FT approached statistical significance (SMD −0.49, 95%CI −0.98 to 0.00), while FT significantly outperformed DTT (SMD −0.90, 95%CI −1.70 to −0.10). Notably, DTT versus CON did not reach statistical significance (SMD −0.38, 95%CI −1.08 to 0.32).

*For stride length*: DTT showed the most prominent effects (SMD −9.03, 95%CI −11.80 to −6.25), followed by ER (SMD −2.40, 95%CI −3.17 to −1.63), CR (SMD −0.73, 95%CI −1.13 to −0.33), and FT (SMD −2.79, 95%CI −3.44 to −2.15). In direct comparisons, DTT significantly outperformed FT (SMD -6.23, 95%CI −9.08 to −3.38), ER (SMD −6.62, 95%CI −9.50 to −3.75), and CR (SMD −8.30, 95%CI −11.10 to −5.50). No significant difference was found between FT and ER (SMD −0.39, 95%CI −1.40 to 0.61), but FT significantly outperformed CR (SMD −2.06, 95%CI −2.82 to −1.31). ER also significantly outperformed CR (SMD −1.67, 95%CI −2.54 to −0.81).

##### 3.3.2.2 Motor function

*For TUG*: All interventions showed significant advantages versus CON (*p* < 0.05). Specifically, CR showed the strongest intervention effect (SMD −11.22, 95%CI −12.87 to −9.57), followed by FT (SMD −11.23, 95%CI −12.99 to −9.46), ER (SMD −10.68, 95%CI −12.37 to −9.00), and DTT (SMD −10.42, 95%CI −12.29 to −8.55). In direct comparisons between interventions, no significant differences were found between CR and DTT (SMD -0.80, 95%CI −1.67 to 0.07), ER (SMD −0.53, 95%CI −0.88 to −0.19), or FT (SMD 0.01, 95%CI −0.62 to 0.63). Similarly, no significant differences were found between DTT and ER (SMD −0.26, 95%CI −1.20 to 0.67) or DTT and FT (SMD −0.81, 95%CI −1.88 to 0.26). Notably, ER versus FT approached statistical significance (SMD -0.54, 95%CI −1.26 to 0.17).

*For UPDRS-III*: Significant differences were found between interventions versus CON (*p* < 0.05). Specifically, DTT (SMD −1.67, 95%CI −2.24 to −1.11) and ER (SMD −0.89, 95%CI −1.27 to −0.51) showed significant advantages versus CON, and CR (SMD −0.79, 95%CI −1.30 to −0.28) also showed statistical significance. In direct comparisons between interventions, DTT significantly outperformed FT (SMD −0.78, 95%CI −1.46 to −0.10) and ER (SMD −0.89, 95%CI −1.64 to −0.13), while no significant differences were found between ER and CR (SMD −0.11, 95%CI -0.74 to 0.53) or FT and DTT (SMD 0.10, 95%CI −0.96 to 1.15). Notably, FT versus CON approached statistical significance (SMD −1.77, 95%CI −2.66 to −0.88). These results are detailed in Table 1.

**Table 1:** Network league table of outcome measures.

Gait velocity				
DTT	−0.28 (−0.97, 0.41)	-0.41 (−1.50, 0.68)	-0.49 (−1.17, 0.20)	−1.28 (−1.92, −0.64)
0.28 (−0.41, 0.97)	ER	−0.13 (−1.05, 0.79)	−0.21 (−0.57, 0.16)	−1.00 (−1.27, −0.73)
0.41 (−0.68, 1.50)	0.13 (−0.79, 1.05)	FT	−0.08 (−0.99, 0.84)	−0.87 (−1.75, 0.01)
0.49 (−0.20, 1.17)	0.21 (−0.16, 0.57)	0.08 (−0.84, 0.99)	CR	−0.79 (−1.04, −0.54)
1.28 (0.64, 1.92)	1.00 (0.73, 1.27)	0.87 (−0.01, 1.75)	0.79 (0.54, 1.04)	CON
Cadence				
ER	−1.56 (−2.31, −0.81)	−2.04 (−2.82, −1.26)	−2.94 (−3.92, −1.97)	−3.33 (−4.01, −2.65)
1.56 (0.81, 2.31)	CR	−0.49 (−0.98, 0.00)	−1.39 (−2.15, −0.62)	−1.77 (−2.08, −1.46)
2.04 (1.26, 2.82)	0.49 (−0.00, 0.98)	FT	−0.90 (−1.70, −0.10)	−1.28 (−1.66, −0.90)
2.94 (1.97, 3.92)	1.39 (0.62, 2.15)	0.90 (0.10, 1.70)	DTT	−0.38 (−1.08, 0.32)
3.33 (2.65, 4.01)	1.77 (1.46, 2.08)	1.28 (0.90, 1.66)	0.38 (−0.32, 1.08)	CON
Stride length				
DTT	−6.23 (−9.08, −3.38)	−6.62 (−9.50, −3.75)	−8.30 (−11.10, −5.50)	−9.03 (−11.80, −6.25)
6.23 (3.38,9.08)	FT	−0.39 (−1.40, 0.61)	−2.06 (−2.82, −1.31)	−2.79 (−3.44, −2.15)
6.62 (3.75, 9.50)	0.39 (−0.61, 1.40)	ER	−1.67 (−2.54, −0.81)	−2.40 (−3.17, −1.63)
8.30 (5.50, 11.10)	2.06 (1.31, 2.82)	1.67 (0.81, 2.54)	CR	−0.73 (−1.13,-0.33)
9.03 (6.25, 11.80)	2.79 (2.15, 3.44)	2.40 (1.63, 3.17)	0.73 (0.33, 1.13)	CON
TUG				
CR	−10.42 (−12.29, −8.55)	−10.68 (−12.37, −9.00)	−11.23 (−12.99, −9.46)	−11.22 (−12.87, −9.57)
10.42 (8.55, 12.29)	DTT	−0.26 (−1.20, 0.67)	−0.81 (−1.88, 0.26)	−0.80 (−1.67, 0.07)
10.68 (9.00, 12.37)	0.26 (−0.67, 1.20)	ER	−0.54 (−1.26, 0.17)	−0.53 (−0.88, −0.19)
11.23 (9.46, 12.99)	0.81 (−0.26, 1.88)	0.54 (−0.17, 1.26)	FT	0.01 (−0.62, 0.63)
11.22 (9.57, 12.87)	0.80 (−0.07, 1.67)	0.53 (0.19, 0.88)	−0.01 (−0.63, 0.62)	CON
UPDRS-III				
FT	−0.10 (−1.15, 0.96)	−0.88 (−1.85, 0.09)	−0.98 (−2.01, 0.04)	−1.77 (−2.66, −0.88)
0.10 (−0.96, 1.15)	DTT	−0.78 (−1.46, −0.10)	−0.89 (−1.64, −0.13)	−1.67 (−2.24, −1.11)
0.88 (−0.09, 1.85)	0.78 (0.10, 1.46)	ER	−0.11 (−0.74, 0.53)	−0.89 (−1.27, −0.51)
0.98 (−0.04, 2.01)	0.89 (0.13, 1.64)	0.11 (−0.53, 0.74)	CR	−0.79 (−1.30, −0.28)
1.77 (0.88, 2.66)	1.67 (1.11, 2.24)	0.89 (0.51, 1.27)	0.79 (0.28, 1.30)	CON

#### 3.3.3 SUCRA rankings

The SUCRA rankings for each outcome measure are presented in Appendix 3.

##### 3.3.3.1 Gait function assessments revealed

For gait velocity, DTT (86.5%) showed optimal efficacy, followed by ER (67.1%) and FT (54.0%), with CR (41.7%) and CON (0.6%) demonstrating weaker effects. For cadence, ER (100.0%) ranked highest, followed by CR (74.4%) and FT (50.2%) with moderate effects, while DTT (22.0%) and CON (3.4%) showed poorer performance. For stride length, DTT (100.0%) exhibited the best outcomes, followed by FT (69.4%) and ER (55.6%), with CR (25.0%) and CON (0.0%) showing limited effects.

##### 3.3.3.2 Motor function assessments indicated

For TUG, CR (100.0%) demonstrated the most significant improvement, followed by DTT (65.3%) and ER (55.5%) with moderate effects, while FT (15.4%) and CON (13.9%) showed suboptimal results. For UPDRS-III, FT (87.6%) ranked highest, closely followed by DTT (85.1%), with ER (42.2%) showing moderate effects and CR (35.2%) and CON (0.0%) demonstrating weaker outcomes.

### 3.4 Risk of bias

**Figure 3:**
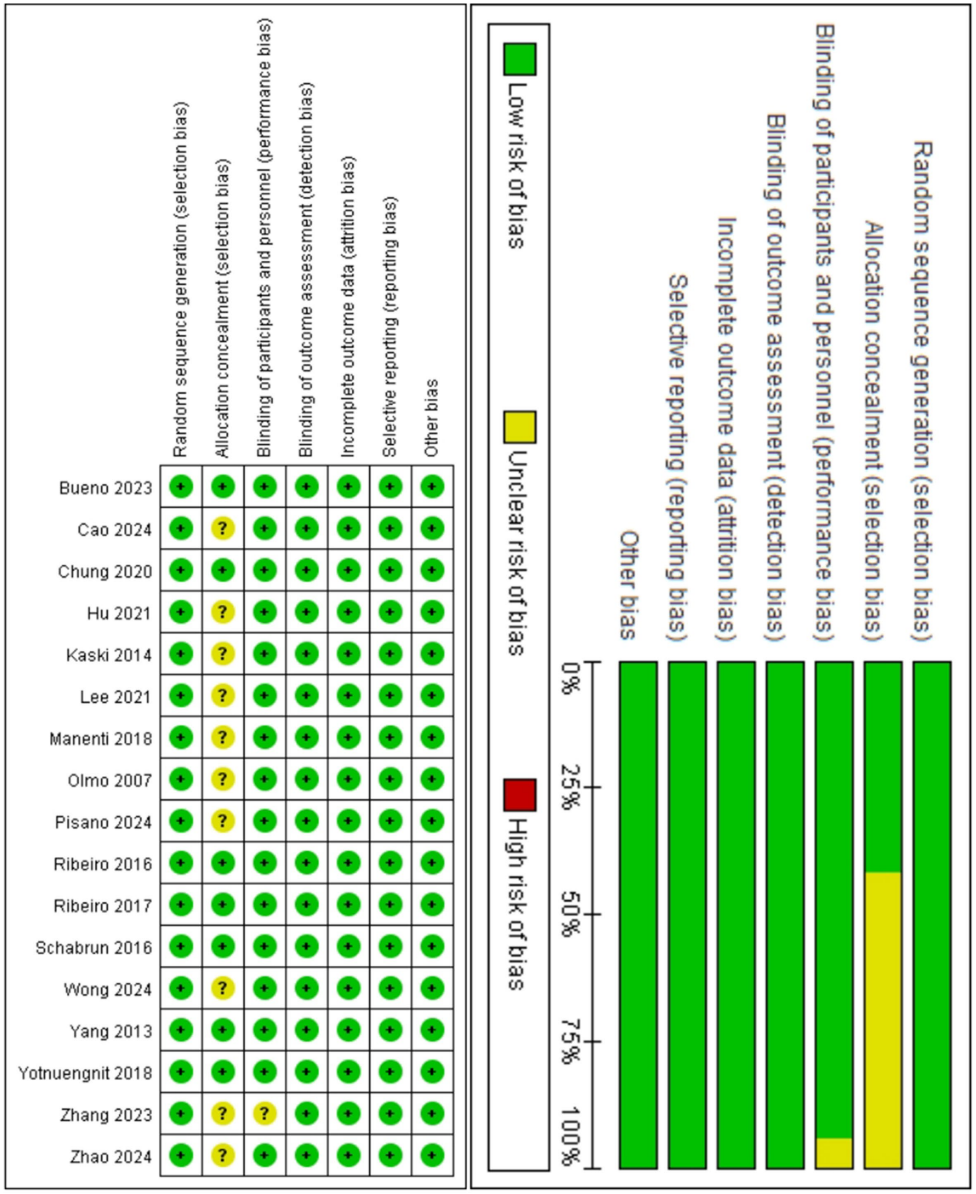
Summary of risk assessment.

Figure 3 demonstrates that among the 17 included studies, the primary source of bias was Selection Bias, particularly unclear allocation concealment methods. Specifically: 10 studies (58.8%) failed to explicitly report allocation concealment procedures.

In overall bias assessment: 16 studies (94.1%) were rated as low risk, indicating high methodological quality and reliable results. Only 1 study (5.9%) was rated as moderate risk due to unreported allocation concealment and lack of blinding description.

To verify result robustness, we conducted sensitivity analysis by excluding this single moderate-risk study. The re-evaluated effect sizes showed no substantial changes in primary conclusions, confirming good stability of findings (see Appendix 4).

### 3.5 Publication bias

A systematic evaluation of publication bias was conducted for the included studies. Most outcome measures showed no evidence of publication bias. However, the symmetry test of the funnel plot for Cadence (Egger’s test: *t* = 2.85, *p* = 0.01) indicated potential publication bias in the study data.

Further sensitivity analysis using the trim-and-fill method for the Cadence measure estimated approximately 8 potentially missing studies. After data imputation, the pooled effect size was adjusted from 0.306 (0.275, 0.337) to 0.223 (0.193, 0.254), suggesting that the potential missing studies had minimal impact on the stability of the overall effect estimate. These results support the reliability of the current meta-analysis conclusions.

The funnel plots are presented in Appendix 5, and the Egger’s test results are shown in Table 2. Appendix 6: Definitions of Rehabilitation Interventions and Parameters of Transcranial Stimulation.

**Table 2:** Egger’s test.

Outcome	Coef.	Std. Em.	*t*	*p*	95% CI	Bias Judgment
Gait velocity	0.45	3.05	0.15	0.88	(−5.89, 6.79)	No significant bias (*p* > 0.05)
Cadence	7.78	2.73	2.85	0.01	(1.93, 13.63)	Potential bias detected (*p* < 0.05)
Stride Length	−1.64	6.91	−0.24	0.82	(−16.69, 13.40)	No significant bias (*p* > 0.05)
TUG	4.44	5.13	0.87	0.40	(−6.73, 15.61)	No significant bias (*p* > 0.05)
UPDRS-III	−4.42	9.65	−0.46	0.66	(−26.26, 17.41)	No significant bias (*p* > 0.05)

## 4 Discussion

### 4.1 Gait function

The results of this study demonstrate that different transcranial stimulation-rehabilitation combinations exert distinct yet complementary effects on gait function in Parkinson’s disease (PD) patients. An integrated analysis of gait parameters reveals a coherent pattern: while both dual-task training (DTT) and exercise-based rehabilitation (ER) significantly improved gait velocity over the control group (CON), suggesting that transcranial stimulation generally potentiates training effects for overall walking speed, each intervention showed unique strengths in specific domains. This aligns with previous findings that transcranial magnetic stimulation (TMS)-combined rehabilitation enhances gait velocity (Doruk et al., 2014), potentially through mechanisms such as enhanced motor cortex excitability (Cholewa et al., 2025). The comparable efficacy in velocity improvement across active interventions, which contrasts with Zhuang et al. (2020) who reported significant between-target differences, may reflect the integrated nature of velocity as an overall gait measure.

The dissociation between cadence and stride length improvements provides mechanistic insight into how different interventions achieve their effects. ER’s optimal efficacy in cadence, significantly outperforming other interventions, likely relates to its emphasis on rhythmic gait training, which directly reinforces cadence regulation mechanisms (Harrison and Earhart, 2023). The rhythmic auditory or cueing components in ER may specifically target temporal gait coordination (Muthukrishnan et al., 2019). Existing evidence supports this cadence-modulating superiority of rhythm-based approaches (Hausdorff et al., 2007), while DTT’s cognitive-motor dual-task paradigm may divert attentional resources from the precise temporal control required for cadence regulation (Mougeot et al., 2016), explaining its relatively weaker effect on this parameter.

Conversely, DTT showed the most pronounced improvement in stride length, significantly surpassing other interventions. This spatial–temporal dissociation suggests DTT may primarily enhance the spatial aspects of gait through mechanisms promoting improved motor planning and integration (Yang et al., 2019). The cognitive demands of dual-tasking may engage neural circuits involved in movement scaling and spatial navigation (Vieira-Yano et al., 2021). Additionally, both FT and ER outperformed CR in stride improvement, suggesting that transcranial stimulation combined with feedback or exercise training may further optimize gait parameters through enhanced sensorimotor integration (Sreenivasan et al., 2023). These findings corroborate previous work demonstrating significant stride length increases with combined interventions (Mo et al., 2024), while meta-analytic evidence confirms exercise therapy’s superior efficacy over pure cognitive training for gait parameters (Xia et al., 2025).

### 4.2 Motor function

For general mobility measured by TUG, all interventions showed significant improvement, with CR showing optimal effects despite no statistically significant between-intervention differences. This pattern suggests that combining transcranial stimulation with conventional physiotherapy may provide comprehensive advantages by integrating broad physiological stimulation with targeted neuromodulation. The comparable efficacy of ER, FT, and DTT implies potential functional equivalence in enhancing mobility, a finding that contextualizes conflicting previous reports in the literature. Grobe et al. reported significant TUG improvement with rTMS-CR combination versus rehabilitation alone (Gaßner et al., 2022), whereas Costa et al. found no difference between tDCS-DTT and CR (Grobe-Einsler et al., 2024), suggesting that patient-specific factors may determine optimal intervention selection.

UPDRS-III results revealed that DTT and ER significantly improved motor symptoms versus CON, with DTT outperforming FT and ER. This pattern indicates that interventions incorporating cognitive-motor integration (DTT) or sustained kinetic engagement (ER) may more effectively address core motor deficits. The superior performance of DTT aligns with demonstrations that TMS-DTT enhances motor-cognitive network synergy for symptom control (Costa-Ribeiro et al., 2021), while findings on DTT’s potential dopaminergic facilitation may explain its broad efficacy (Potvin-Desrochers et al., 2023). Notably, while FT did not show universal superiority across all measures, its high SUCRA ranking for specific motor domains corresponds with reports of FT’s unique benefits for postural stability (Johansson et al., 2023), highlighting the importance of outcome measure selection in evaluating intervention efficacy (Raethjen et al., 2020).

## 5 Limitation

This study has two main limitations. First, the patient characteristics did not include the age of symptom onset—a factor that may correlate with disease progression and differential responses to intervention, preventing further analysis of how age-related variables potentially influence intervention outcomes. Second, no data on the angles of the feet, ankles, or legs during walking were provided; such biomechanical parameters are critical for determining whether improvements in balance and posture translate to enhanced control over the center of gravity, thus limiting the sufficiency of verifying the intervention mechanism related to center-of-gravity control. This study is limited by the incomplete reporting of the Chinese search strategy, which may affect reproducibility. Additionally, restricting inclusion to Chinese and English publications could introduce language bias.

## 6 Conclusion

This NMA demonstrates that transcranial stimulation combined with rehabilitation effectively improves gait and motor function in Parkinson’s disease, with differential effects across interventions. Clinically, DTT shows superior efficacy for stride length and motor symptoms, while ER optimally improves cadence. CR combined with stimulation provides comprehensive motor benefits. These findings support personalized therapy selection: DTT for patients with stride deficits/freezing, ER for those with festination, and CR for general mobility improvement. The robust treatment effects and low heterogeneity enhance clinical applicability. Future studies should standardize protocols to minimize allocation bias. These results provide evidence-based guidance for optimizing neurorehabilitation strategies in PD management.

## Data Availability

The original contributions presented in the study are included in the article/Supplementary material, further inquiries can be directed to the corresponding author.
