# Supplementary material for: Transcranial stimulation combined with four rehabilitation therapies for gait and motor function in Parkinson’s disease: a network meta-analysis of 23 RCTs
Source: Front Aging Neurosci. 2025 Dec 15;17:1670825. doi: 10.3389/fnagi.2025.1670825 (PMC12745421; doi:10.3389/fnagi.2025.1670825)

**Appendix 1.** Characteristics of included literature

| study | country | Intervention category | Sample size(F/M) | Age(mean±SD) | Intervention frequency | Intervention duration | Cognition | PD disease progression (years) |
| --- | --- | --- | --- | --- | --- | --- | --- | --- |
| Zhao 2024 [1] | China | CON | 24(11/13) | 68.58±10.05 | 30min,twice/day,5 times/week | 4week | 23.21±2.67 | 4.96±1.55 |
|  |  | CON | 24(10/14) | 67.92±6.82 | 30min,twice/day,5 times/week |  | 22.29±2.074 | 5.17±1.74 |
|  |  | FT | 24(8/16) | 68.88±7.73 | 30min,twice/day,5 times/week |  | 23.08±2.93 | 5.04±1.73 |
| Yotnuengnit 2018 [2] | Thailand | CON | 18(8/10) | 64.4+7.8 | 30min, 3 times/week | 2week |  | 7.9+3.9 |
|  |  | CR | 17(6/11) | 68.2+9.8 | 30min+30min, 3 times/week |  |  | 9.4+5.3 |
|  |  | CON | 18(6/12) | 62.7+8.8 | 30min, 3 times/week |  |  | 6.6+3.6 |
| Bueno 2023 [3] | Brazil | CON | 13(5/8) | 66.38 ±10.24 | 20 | 1week | 27.77 ±2.16 | 4.75±6.15 |
|  |  | CON | 13(5/8) | 69.62 ±6.22 | 30 |  | 27.15 ±2.47 | 6.59 ±5.29 |
|  |  | CR | 12(5/7) | 63.92 ±11.87 |  |  | 25.50 ±3.92 | 5.11 ±4.06 |
|  |  | CR | 12(3/9) | 60.75 ±10.63 |  |  | 28.25 ±1.96 | 5.16 ±4.13 |
| Hu2021 [4] | China | CR | 49(19/30) | 64.23±4.78 | 45min, 5 times/week | 12week | 23.65±1.79 | 33.02±10.65 |
|  |  | CON | 49(21/28) | 63.68±5.22 | 45min, 5 times/week |  | 24. 21±2. 16 | 32.32±12.44 |
| Wong 2024 [5] | China | ER | 17(8/9) | 68.1+5.8 | 50min,2-3 times/week | 5week | 27.3+3.3 | 5.5+4.3 |
|  |  | CON | 17(12/5) | 66.8+6.9 | 50min,2-3 times/week |  | 27.81+2.6 | 7.9+5.6 |
| Olmo 2007 [6] | Spain | CR | 8(5/3) | 61.7±5.22 | 30min,once/day | 10days |  |  |
|  |  | CON | 5(3/2) | 61.7±5.22 | 30min,once/day |  |  |  |
| Yang 2013 [7] | China | ER | 10(5/5) | 65.20 ± 11.08 | 40min, 3 times/week | 4week | 28.40±2.12 | 6.40 ± 2.76 |
|  |  | CON | 10(3/7) | 67.00 ± 13.21 | 40min, 3 times/week |  | 29.50 ± 0.97 | 6.35 ± 3.58 |
| Chung 2020 [8] | China | ER | 17(7/10) | 62.7+6.8 | 30min, 3 times/week | 3week |  | 5.2+3.4 |
|  |  | ER | 17(8/9) | 62.1+5.7 | 30min, 3 times/week |  |  | 7.5+4.9 |
|  |  | CON | 16(9/7) | 62.1+5.7 | 30min, 3 times/week |  |  | 6.9+3.3 |
| Kaski 2014 [9] | Argentina | CR | 8 |  | 30min, twice/week | 2week |  |  |
|  |  | CON | 8 |  | 30min, twice/week |  |  |  |
|  |  | CON | 8 |  | 30min, twice/week |  |  |  |
|  |  | CON | 8 |  | 30min, twice/week |  |  |  |
| Cao 2024 [10] | China | ER | 27(13/14) | 66.59 ± 7.07 | 30min, 6 times/week | 2week | 25.26 ± 1.65 | 3.00+2.50 |
|  |  | CON | 27(15/12) | 67.19 ± 7.44 | 30min, 6 times/week |  | 24.81 ± 1.59 | 5.00+2.75 |
| Ribeiro 2017 [11] | Brazil | FT | 11(3/8) | 61.1±9.1 | 30min, 3 times/week | 4week | 26.4±3.0 | 6.1±3.8 |
|  |  | CON | 11(4/7) | 62.0±16.7 | 30min, 3 times/week |  | 25.7±4.9 | 6.3±3.7 |
| Ribeiro 2016 [12] | Brazil | FT | 11(3/8) | 61.1±9.1 | 43min, 3 times/week | 4week | 26.4±3.0 | 6.1±3.8 |
|  |  | CON | 11(4/7) | 62.0±16.7 | 43min, 3 times/week |  | 25.7±4.9 | 6.3±3.7 |
| Lee 2021 [13] | South Korea | FT | 15(9/6) | 70.00+3.76 | 50min, 5 times/week | 4week | 26.33+1.35 | 6.27+1.03 |
|  |  | CON | 15(7/8) | 71.33+3.27 | 50min, 5 times/week |  | 26.87+1.51 | 7.00+1.41 |
| Schabrun 2016 [14] | Australia | DTT | 8(0/8) | 72±4.9 | 60min, 3 times/week | 3week | 29.0±0.76 | 6.9±4.4 |
|  |  | CON | 8(6/2) | 63±11.0 | 60min, 3 times/week |  | 29.7±0.46 | 4.6±3.9 |
| Pisano 2024 [15] | Italy | DTT | 9(6/3) | 71 ± 8.6 | 20min, 5 times/week | 10days |  |  |
|  |  | CON | 8(4/4) | 65.3 ± 8.5 | 20min, 5 times/week |  |  |  |
| Zhang 2023 [16] | China | ER | 32(13/19) | 63.87 ± 5.60 | 40min, 5 times/week | 8week |  | 4.23 ± 0.79 |
|  |  | CON | 32(15/17) | 64.03 ± 5.28 | 50min, 5 times/week |  |  | 4.50 ± 0.82 |
| Manenti 2018 [17] | Italy | DTT | 11(6/5) | 65.5 ± 6.4 | 50min, 5 times/week | 2week |  | 6.2 ± 3.9 |
|  |  | CON | 11(4/7) | 63.8 ± 7.1 | 50min, 5 times/week |  |  | 7.6 ± 3.4 |

1. Zhao Weijing, Li Yongping, You Hong, Feng Xianhui & Lei Yanli. (2024). The effect of repetitive transcranial magnetic stimulation combined with visual feedback balance training on balance and gait in patients with Parkinson's disease. Chinese Journal of Rehabilitation Medicine, 39 (09), 1327-1331.
2. Yotnuengnit, P., Bhidayasiri, R., Donkhan, R., Chaluaysrimuang, J., & Piravej, K. (2018). Effects of Transcranial Direct Current Stimulation Plus Physical Therapy on Gait in Patients With Parkinson Disease: A Randomized Controlled Trial. American journal of physical medicine & rehabilitation, 97(1), 7–15. <https://doi.org/10.1097/PHM.0000000000000783>
3. Bueno, M. E. B., Silva, T. C. O. D., de Souza, R. J., Volpe, R. P., Moura, F. A., & Smaili, S. M. (2023). Acute effects of transcranial direct current stimulation combined with physical therapy on the balance and gait in individuals with Parkinson's disease: A randomized controlled trial. Clinical neurology and neurosurgery, 226, 107604. <https://doi.org/10.1016/j.clineuro.2023.107604>
4. Hu, X., Xue, C., & Liu, Z. (2021). The effect of transcranial direct current stimulation-assisted functional rehabilitation training on the rehabilitation of patients with Parkinson's disease. Chinese Journal of Geriatrics, 41(17), 3724-3727.
5. Wong, P. L., Yang, Y. R., Huang, S. F., & Wang, R. Y. (2024). Effects of DLPFC tDCS Followed by Treadmill Training on Dual-Task Gait and Cortical Excitability in Parkinson's Disease: A Randomized Controlled Trial. Neurorehabilitation and neural repair, 38(9), 680–692. <https://doi.org/10.1177/15459683241268583>
6. del Olmo, M. F., Bello, O., & Cudeiro, J. (2007). Transcranial magnetic stimulation over dorsolateral prefrontal cortex in Parkinson's disease. Clinical neurophysiology : official journal of the International Federation of Clinical Neurophysiology, 118(1), 131–139. <https://doi.org/10.1016/j.clinph.2006.09.002>
7. Yang, Y. R., Tseng, C. Y., Chiou, S. Y., Liao, K. K., Cheng, S. J., Lai, K. L., & Wang, R. Y. (2013). Combination of rTMS and treadmill training modulates corticomotor inhibition and improves walking in Parkinson disease: a randomized trial. Neurorehabilitation and neural repair, 27(1), 79–86. <https://doi.org/10.1177/1545968312451915>
8. Chung, C. L., Mak, M. K., & Hallett, M. (2020). Transcranial Magnetic Stimulation Promotes Gait Training in Parkinson Disease. Annals of neurology, 88(5), 933–945. <https://doi.org/10.1002/ana.25881>
9. Kaski, D., Dominguez, R. O., Allum, J. H., Islam, A. F., & Bronstein, A. M. (2014). Combining physical training with transcranial direct current stimulation to improve gait in Parkinson's disease: a pilot randomized controlled study. Clinical rehabilitation, 28(11), 1115–1124. <https://doi.org/10.1177/0269215514534277>
10. Cao Wenhui, Lin Ge Wei, Xu Ying, & Yang Xiaofan. (2024). The effect of high-frequency repetitive transcranial magnetic stimulation combined with aerobic exercise training on motor disorders in patients with mild to moderate Parkinson's disease. Chinese Journal of Geriatric Cardiovascular Diseases, 26(11), 1338-1342.
11. Costa-Ribeiro, A., Maux, A., Bosford, T., Aoki, Y., Castro, R., Baltar, A., Shirahige, L., Moura Filho, A., Nitsche, M. A., & Monte-Silva, K. (2017). Transcranial direct current stimulation associated with gait training in Parkinson's disease: A pilot randomized clinical trial. Developmental neurorehabilitation, 20(3), 121–128. <https://doi.org/10.3109/17518423.2015.1131755>
12. Costa-Ribeiro, A., Maux, A., Bosford, T., Tenório, Y., Marques, D., Carneiro, M., Nitsche, M. A., Filho, A. M., & Monte-Silva, K. (2016). Dopamine-independent effects of combining transcranial direct current stimulation with cued gait training on cortical excitability and functional mobility in Parkinson's disease. Journal of rehabilitation medicine, 48(9), 819–823. <https://doi.org/10.2340/16501977-2134>
13. Lee, S. A., & Kim, M. K. (2021). The Effect of Transcranial Direct Current Stimulation Combined with Visual Cueing Training on Motor Function, Balance, and Gait Ability of Patients with Parkinson's Disease. Medicina (Kaunas, Lithuania), 57(11), 1146. <https://doi.org/10.3390/medicina57111146>
14. Schabrun, S. M., Lamont, R. M., & Brauer, S. G. (2016). Transcranial Direct Current Stimulation to Enhance Dual-Task Gait Training in Parkinson's Disease: A Pilot RCT. PloS one, 11(6), e0158497. <https://doi.org/10.1371/journal.pone.0158497>
15. Pisano, F., Mellace, D., Fugatti, A., Aiello, E. N., Diotti, S., Curti, B., Giust, A., Marfoli, A., Perin, C., De Sandi, A., Alimonti, D., Priori, A., & Ferrucci, R. (2024). Cerebellar tDCS combined with augmented reality treadmill for freezing of gait in Parkinson's disease: a randomized controlled trial. Journal of neuroengineering and rehabilitation, 21(1), 173. <https://doi.org/10.1186/s12984-024-01457-z>
16. Zhang, L., Gao, L., Xue, C., Zhao, Y., Zhao, L., & Liu, A. (2023). Clinical efficacy of virtual reality technology combined with repetitive transcranial magnetic stimulation in the treatment of mild cognitive impairment in Parkinson's disease. Chinese Journal of Rehabilitation, 38(03), 148-152.
17. Manenti, R., Cotelli, M. S., Cobelli, C., Gobbi, E., Brambilla, M., Rusich, D., Alberici, A., Padovani, A., Borroni, B., & Cotelli, M. (2018). Transcranial direct current stimulation combined with cognitive training for the treatment of Parkinson Disease: A randomized, placebo-controlled study. Brain stimulation, 11(6), 1251–1262. <https://doi.org/10.1016/j.brs.2018.07.046>

**Appendix 2.** Pairwise meta-analysis forest plots of outcome measures


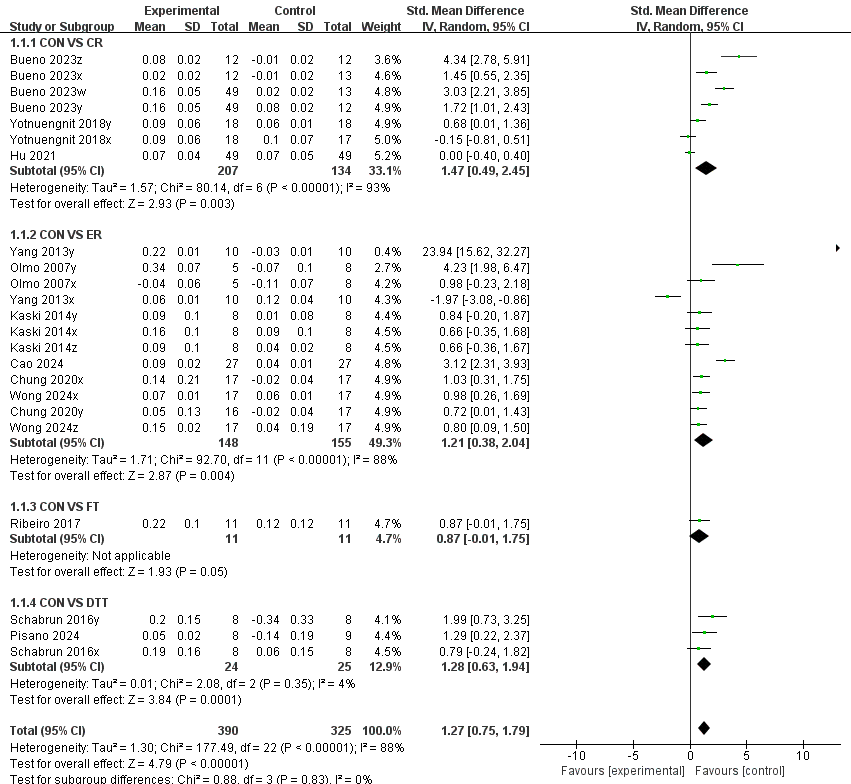


**Appendix 2-1.** Pairwise meta-analysis forest plots of Gait velocity


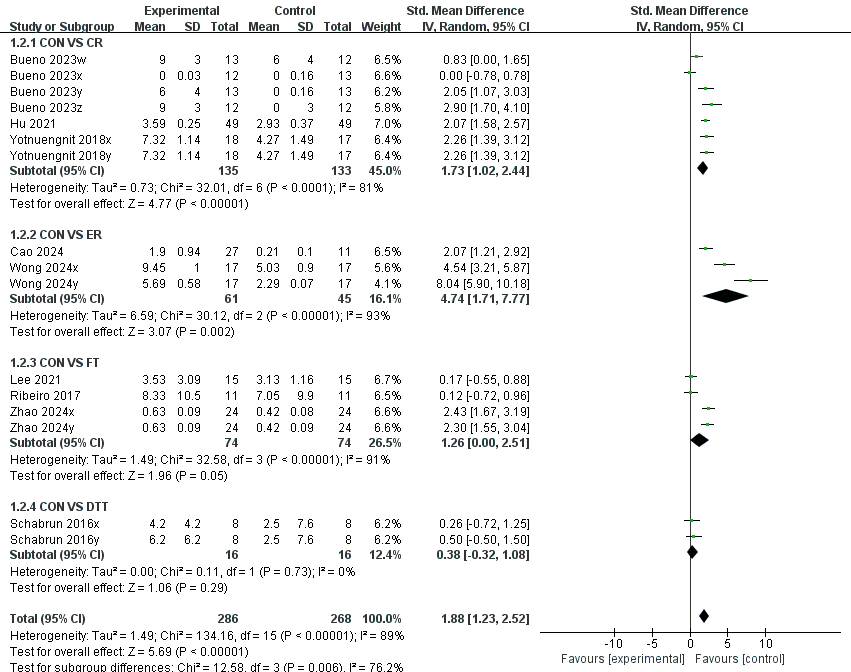


**Appendix 2-2.** Pairwise meta-analysis forest plots of Cadence


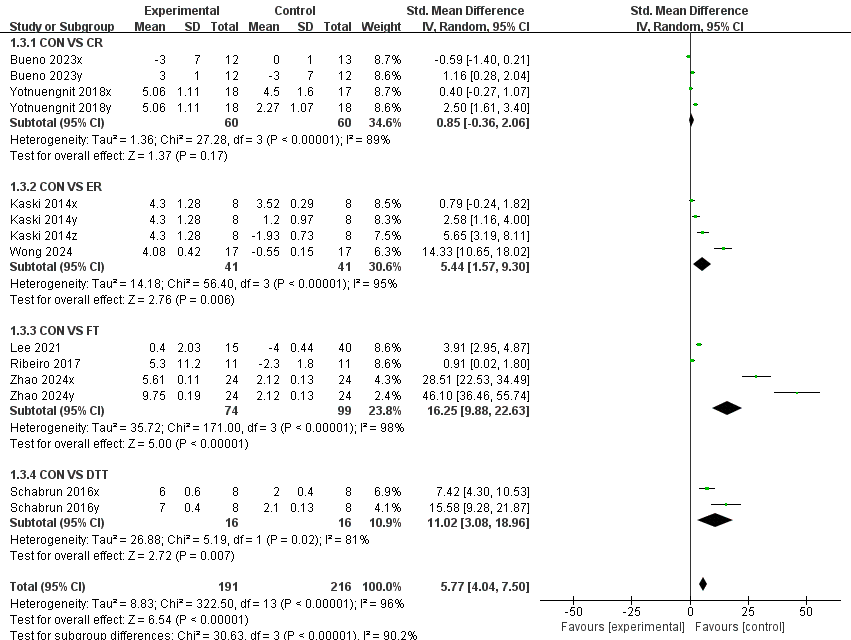


**Appendix 2-3.** Pairwise meta-analysis forest plots of Stride Length


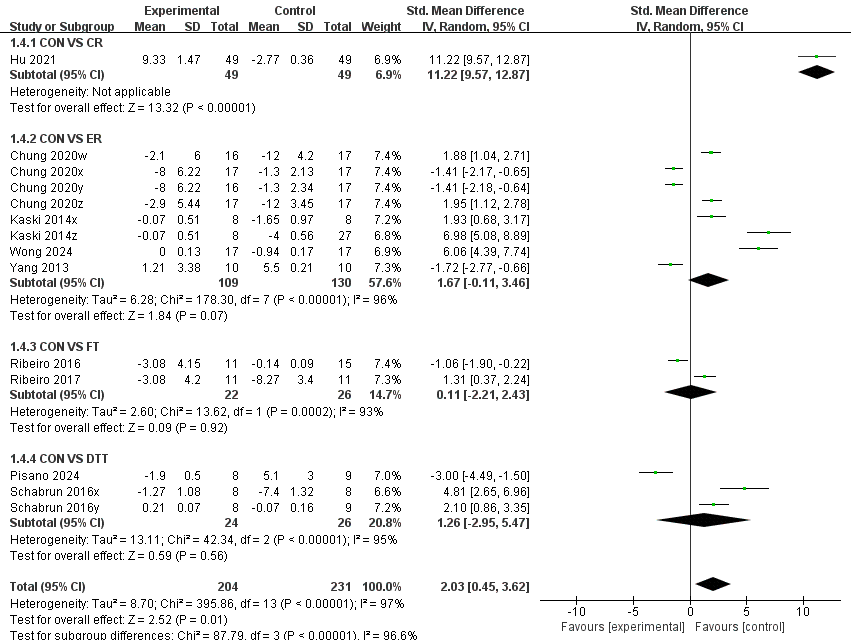


**Appendix 2-4.** Pairwise meta-analysis forest plots of TUG


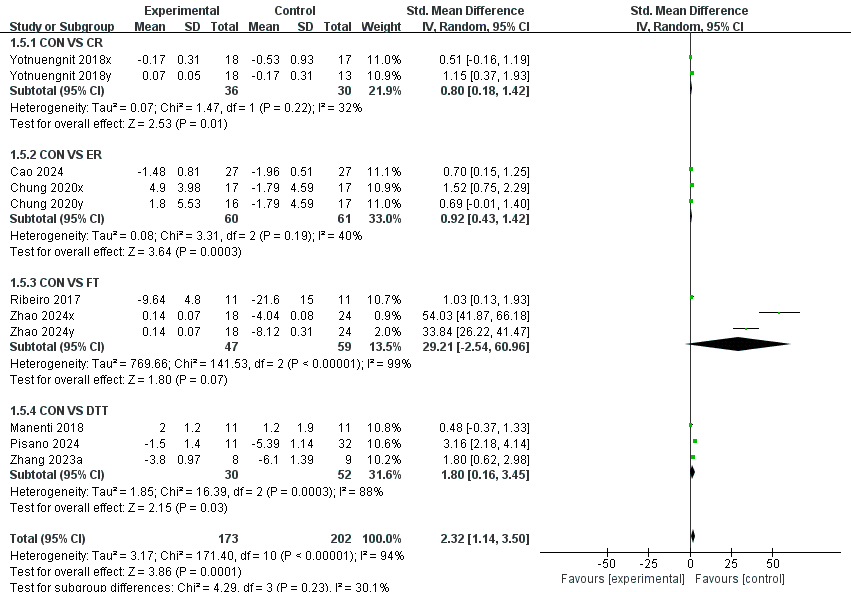


**Appendix 2-5.** Pairwise meta-analysis forest plots of UPDRS-III

**Appendix 3.** SUCRA ranking plots of outcome measures


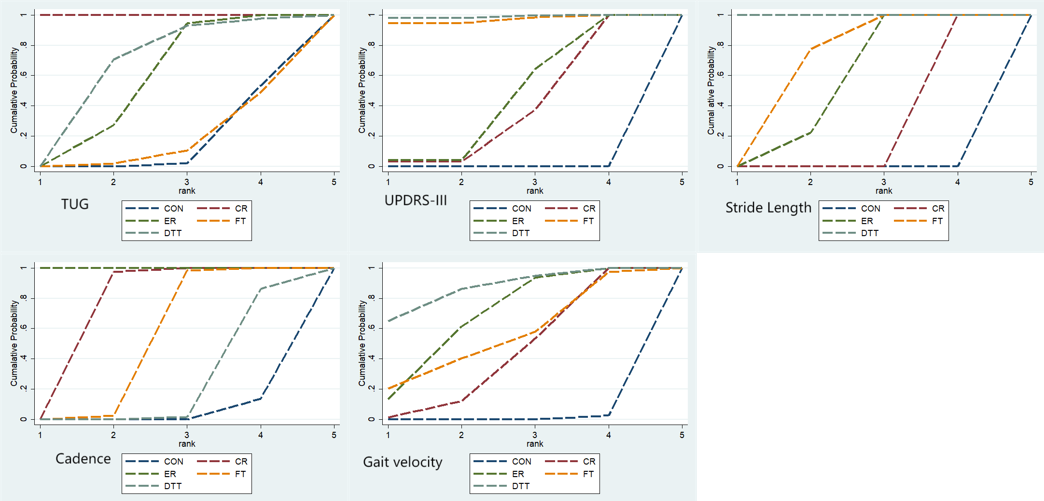


**Appendix 4.** Forest plots after exclusion of studies with moderate risk of bias


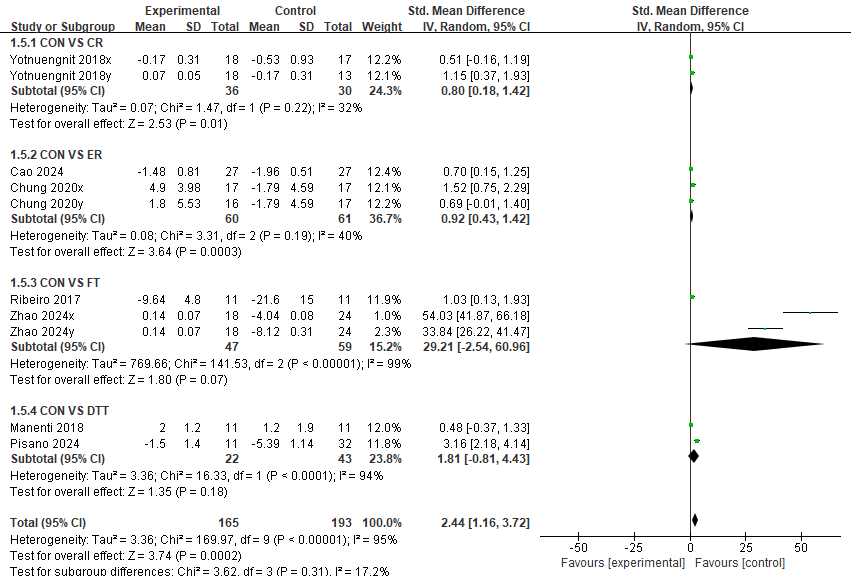


**Appendix 5.** Adjusted comparison funnel plots of outcome measures


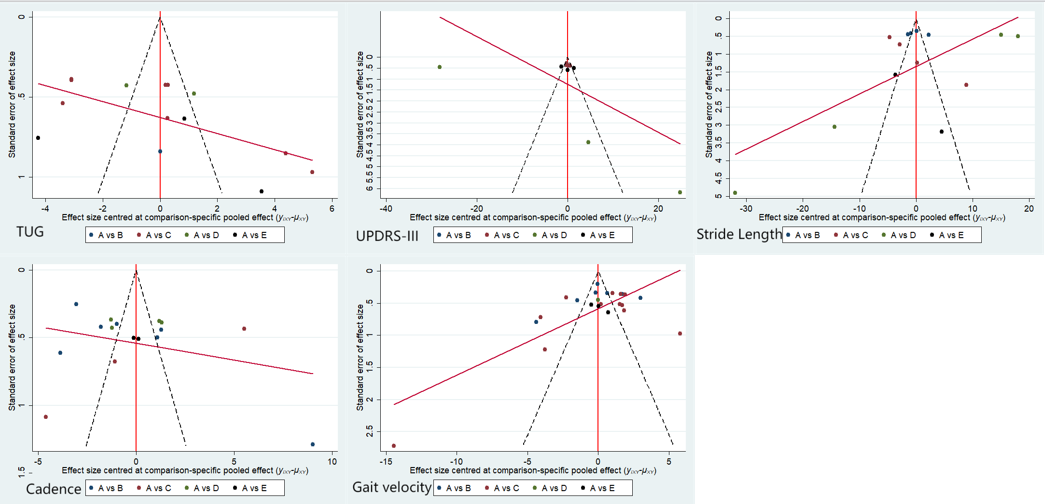

Supplement: Supplementary file 1 [file Table_1.DOCX]
