# Supplementary material for: Transcranial stimulation combined with four rehabilitation therapies for gait and motor function in Parkinson’s disease: a network meta-analysis of 23 RCTs
Source: Front Aging Neurosci. 2025 Dec 15;17:1670825. doi: 10.3389/fnagi.2025.1670825 (PMC12745421; doi:10.3389/fnagi.2025.1670825)
Supplement: Supplementary file 2 [file Table_2.DOCX]

**Appendix 6. Definitions of Rehabilitation Interventions and Parameters of Transcranial Stimulation**

| **Category** | **Operational Definition** | **Stimulation Parameters** | **Control Group Composition** |
| --- | --- | --- | --- |
| **Conventional Rehabilitation (CR)** | Standard physical therapy focusing on balance, coordination, and functional mobility training, often including stretching, strengthening, and gait practice. | **tDCS**: 1–2 mA, M1 region, 20–30 min, 1–5 sessions/week **rTMS**: 5–20 Hz, M1/premotor cortex, 1000–2000 pulses/session, 80–120% MT | Rehabilitation training alone / Sham stimulation + rehabilitation / Transcranial stimulation alone |
| **Exercise Rehabilitation (ER)** | Structured aerobic or resistance exercise programs (e.g., treadmill training, cycling, strength training) aimed at improving cardiovascular fitness and motor performance. | **tDCS**: 1–2 mA, M1/DLPFC, 20–30 min, 1–5 sessions/week **rTMS**: 5–20 Hz, M1/cerebellum, 1000–2000 pulses/session, 80–120% MT | Rehabilitation training alone / Sham stimulation + rehabilitation / Transcranial stimulation alone |
| **Feedback Training (FT)** | Use of visual, auditory, or proprioceptive feedback (e.g., mirrors, biofeedback devices) to enhance motor control and postural adjustment. | **tDCS**: 1–2 mA, M1/PFC, 20–30 min, 1–5 sessions/week **rTMS**: 1–10 Hz, M1/SMA, 1000–2000 pulses/session, 80–120% MT | Rehabilitation training alone / Sham stimulation + rehabilitation / Transcranial stimulation alone |
| **Dual-Task Training (DTT)** | Concurrent performance of motor and cognitive tasks (e.g., walking while counting, obstacle negotiation with arithmetic) to improve multitasking ability. | **tDCS**: 1–2 mA, M1/DLPFC, 20–30 min, 1–5 sessions/week **rTMS**: 5–20 Hz, M1/PFC, 1000–2000 pulses/session, 80–120% MT | Rehabilitation training alone / Sham stimulation + rehabilitation / Transcranial stimulation alone |

**Abbreviations:**
tDCS: transcranial direct current stimulation; rTMS: repetitive transcranial magnetic stimulation;
M1: primary motor cortex; DLPFC: dorsolateral prefrontal cortex; SMA: supplementary motor area;
MT: motor threshold; mA: milliampere; Hz: hertz.

**Note:**

- “Shock stimulation” refers to **sham/placebo stimulation** where devices mimic real stimulation without delivering active current or magnetic fields.
- Control groups included one or more of the following: rehabilitation alone, sham stimulation combined with rehabilitation, or transcranial stimulation alone.
